# Comparison of Standard and Prone‐Position Electrocardiograms in COVID‐19 Patients With Pulmonary Complications: Correlations and Implications

**DOI:** 10.1002/clc.70024

**Published:** 2024-09-30

**Authors:** Pattarapong Makarawate, Krissanachai Chimtim, Thapanawong Mitsungnern, Pariwat Phungoen, Supap Imoun, Piroon Mootsikapun, Thanat Tangpaisarn, Praew Kotruchin

**Affiliations:** ^1^ Department of Internal Medicine, Faculty of Medicine Khon Kaen University Khon Kaen Thailand; ^2^ Department of Emergency Medicine, Faculty of Medicine Khon Kaen University Khon Kaen Thailand; ^3^ Accident and Emergency Stroke Unit, Srinagarind Hospital, Faculty of Medicine Khon Kaen University Khon Kaen Thailand

**Keywords:** ARDS, Asian, COVID‐19, electrocardiogram, electrophysiology, pneumonia, prone

## Abstract

**Background:**

Previous research highlighted variability in electrocardiogram (ECG) readings across patient positions, particularly in the context of COVID‐19 patients with pulmonary complications requiring prone positioning as part of the treatment.

**Objective:**

This study aimed to elucidate the effects of prone positioning on ECG parameters and explore its association with the severity of COVID‐19.

**Methods:**

A prospective cohort study involved 60 patients diagnosed with COVID‐19 and presenting pulmonary complications. ECGs were recorded in both supine and prone positions, and analyzed for various parameters including heart rate, QRS axis, and QTc interval. Clinical severity was assessed using APACHE II scores and SpO_2_/FiO_2_ ratios.

**Results:**

Prone positioning led to an increase in heart rate (mean difference: 2.100, 95% CI: 0.471–3.729, *p* = 0.012), with minor shifts in the QRS axis. Heart rate and QRS axis demonstrated strong positive correlations between positions, with Pearson's correlation coefficients of 0.927 and 0.894, respectively. The study also found a significant association between prolonged QTc intervals in the prone position and elevated APACHE II scores, with a relative risk of 10.75 (95% CI: 1.82–63.64, *p* = 0.008).

**Conclusions:**

The prone positioning caused minor yet significant changes in heart rate and QRS axis. The correlation of prolonged QTc intervals in the prone position with higher APACHE II scores suggests the prognostic relevance of prone ECG in COVID‐19 patients. However, further research is needed to fully understand the clinical implications and mechanisms of these findings.

## Graphical abstract

This study enrolled 60 patients diagnosed with COVID‐19 and pulmonary complications. The study compares ECG parameters in the supine versus prone positions. Key ECG measurements include heart rate (HR), QRS axis, P wave axis, PR interval, QRS duration, and QTc interval. *In the prone position*, a significant increase in HR was observed compared to the supine ECG. There is an association between HR and the severity of COVID‐19, namely, the SF ratio (< 300), with an odds ratio (OR) of 0.96 (95% CI: 0.93–0.99). Similarly, a significant association exists between ST‐segment changes and an APACHE II score greater than 12, with an OR of 6.52 (95% CI: 1.24–34.28).

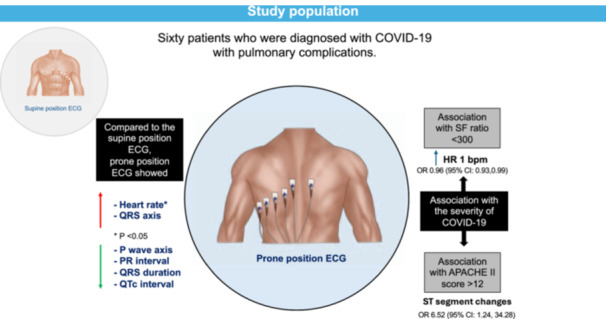

## 1 Introduction

The initial documentation of alterations in 12‐lead electrocardiogram (ECG) readings when subjects were in a prone position dates back to 1992. In this study, a quarter of the ECGs recorded from healthy volunteers were erroneously interpreted as indicative of ischemic conditions [1]. After its initial discovery, the prone 12‐lead ECG (p12‐ECG) garnered renewed interest during the Coronavirus disease 2019 (COVID‐19) pandemic [2].

A clinical review of COVID‐19 indicates that 5% of infections, or 20% of hospitalized cases, present with severe symptoms necessitating intensive care, with over 75% requiring supplemental oxygen [3]. The major complications of COVID‐19 are pneumonia, affecting 75% of cases, and acute respiratory distress syndrome (ARDS), seen in 15% [3]. This highlights the importance of protocols for managing acute hypoxic respiratory failure (AHRF) and ARDS, including prone positioning. This technique enhances arterial oxygenation and diminishes shunt effects, without altering cardiac output, pH, partial pressure of carbon dioxide (PaCO_2_), functional residual capacity, or pulmonary vascular driving pressure, compared to the supine position [4, 5, 6]. Consequently, the 2023 ARDS guidelines recommend prone positioning for moderate to severe COVID‐19 ARDS cases with an arterial oxygen partial pressure (PaO_2_)/fractional inspired oxygen (FiO_2_), PF ratio < 150 mmHg. This includes extended prone sessions of 16 h or more for patients with persistent PF ratio < 150 mmHg to lower mortality rate. Additionally, awake‐prone positioning is advised for non‐intubated patients with COVID‐19‐related AHRF to decrease the need for intubation, establishing it as a standard treatment protocol [7].

In COVID‐19 AHRF/ARDS patients, cardiovascular complications such as myocardial injury, myocarditis, acute myocardial infarction (AMI), heart failure, dysrhythmias, and venous thromboembolic events are notable. Approximately 25% of COVID‐19 patients experience AMI, potentially due to direct and indirect myocardial damage, leading to both type I and II myocardial infarctions, even in those without previous cardiovascular comorbidities [8, 9, 10, 11, 12, 13, 14]. Additionally, pre‐existing cardiovascular diseases significantly elevate the risk of adverse clinical outcomes [9, 12]. Consequently, vigilant cardiac monitoring is essential for the early detection of cardiac injuries and for predicting disease‐related complications. However, conducting ECGs in prone‐positioned patients poses challenges regarding the reliability of the readings. Due to these challenges, the prone ECG has become a focal point of study. In 2020, two case reports highlighted abnormal prone ECG patterns in COVID‐19 patients, characterized by low amplitudes and pronounced Q waves in leads V1 to V3, resembling an antero‐septal infarct. Remarkably, these anomalies reverted to normal upon repositioning the patients to a supine position [15, 16]. A subsequent prospective‐multicenter study with healthy volunteers attributed these ECG changes to increased impedances from intervening lung tissues and the extended distance between the precordial electrodes of the prone ECG and the center of the heart [17]. Furthermore, a single‐center study involving healthy volunteers reported that prone ECGs, when excluding the V1‐V3 precordial leads, were largely in agreement with standard or supine ECGs, particularly in terms of QRS and QT interval morphology in leads II and V6 [18]. Conversely, another study in a similar cohort reported significant variations in heart rate (HR), QRS axis, amplitude, duration, morphology, and corrected QT interval (QTc) [19]. However, it is important to note that the findings from these studies are not directly transferable to patients with COVID‐19 and AHRF/ARDS.

A study focusing on ECG monitoring utilized frontal plane leads and V6 to compare ECG in both supine and prone positions in 22 COVID‐19 ARDS patients, 45% of whom had structural or ischemic heart comorbidities. The findings revealed a significant increase in PR duration and a decrease in QRS duration in the prone‐position ECG, without notable changes in the QT interval. There were excellent correlations observed between the QRS axis, PR, RR, QRS, and QT intervals [20]. These results suggest that ECG can be reliably monitored in the prone position using four electrodes repositioned to the back, however, the evidence comparing the prone‐position 12‐lead ECG with the standard 12‐lead ECG in moderate to severe COVID‐19 AHRF patients is scarce [21]. Therefore, we aimed to elucidate the relationship between ECG parameters obtained from a prone position and a supine position in the context of COVID‐19.

## 2 Materials and Methods

### 2.1 Study Design and Population

This was a prospective cohort study (August 3–November 10, 2023) conducted within the specialized COVID‐19 treatment unit of Srinagarind Hospital, Khon Kaen University, Thailand. The unit is equipped with negative pressure chambers and isolation wards, catering specifically to COVID‐19 patients with pulmonary complications. The Khon Kaen University Ethics Committee for Human Research approved the study protocol (No. HE641442). This study was conducted following the ethical standards laid down in the 1964 Declaration of Helsinki and its later amendments.

#### 2.1.1 Study Population

The study cohort comprised individuals diagnosed with COVID‐19 exhibiting pulmonary complications admitted to the COVID‐19 ward of Srinagarind Hospital. Eligibility criteria encompassed individuals aged 18 years or older who were prescribed prone positioning for lung recruitment by a pulmonary specialist. Exclusion criteria were as follows: patients undergoing mechanical ventilation, and patients for whom electrocardiography (ECG) was unfeasible due to anatomical anomalies or superficial dermatological conditions.

#### 2.1.2 Objectives

The primary objective of this study was to elucidate the relationship between ECG parameters obtained from a prone position (prone ECG) and a supine position (standard ECG) in the context of COVID‐19.

The secondary objective focused on comparing clinical diagnoses inferred from ECG interpretations by cardiologists, thereby evaluating the diagnostic utility of prone ECG versus standard ECG in a clinical setting.

### 2.2 Data Collection

Data acquisition commenced postinformed consent, which was duly signed by each patient. After consent, vital signs were meticulously measured, and blood specimens were procured in strict adherence to the prevailing standard care protocols for COVID‐19 patients at the hospital. Management of each case was conducted in collaboration with a specialist in infectious and pulmonary diseases. Therapeutic interventions, including prescribed medications, oxygen therapy, and airway management, along with comprehensive clinical data throughout hospitalization, were systematically retrieved from the electronic medical records system (Health Object). Following the patient's settlement into the assigned bed, the ECGs were performed per the delineated methodology by using Philips PageWriter TC20 (USA) with the paper speed setting of 25 mm/s, and the amplitude setting of 10 mm/mV.

### 2.3 Operating Definition

#### 2.3.1 COVID‐19 Diagnosis

COVID‐19 was diagnosed by a positive test by polymerase chain reaction (PCR) for the presence of the SARS‐CoV‐2 virus from specimens, such as nasopharyngeal or oropharyngeal swabs [2].

#### 2.3.2 Pulmonary Complications

Pulmonary complications in COVID‐19 patients refer to a range of respiratory disorders and abnormalities that arise because of infection with the SARS‐CoV‐2 virus [3], including symptoms such as persistent cough, shortness of breath, and respiratory distress that are key clinical indicators of pulmonary involvement with either of the positive test below.

− Chest X‐ray (CXR) interpreted by a radiologist revealed lung abnormalities such as consolidation or ground‐glass opacities.
− Computed tomography (CT) scan detected early, or mild forms of lung involvement such as ground‐glass opacities and other patterns of lung damage seen in COVID‐19.
− Arterial blood gas (ABG) analysis revealed hypoxemia and/or hypoventilation.
− Elevated levels of C‐reactive protein (CRP), and D‐dimer suggested lung involvement and severity of inflammation.

### 2.4 Outcome Measures

#### 2.4.1 ECG Acquisition

The method for prone position ECG was designed to ensure that the prone ECG was acquired systematically, allowing for a reliable comparison between the standard and prone ECG recordings.

**Figure 1:**
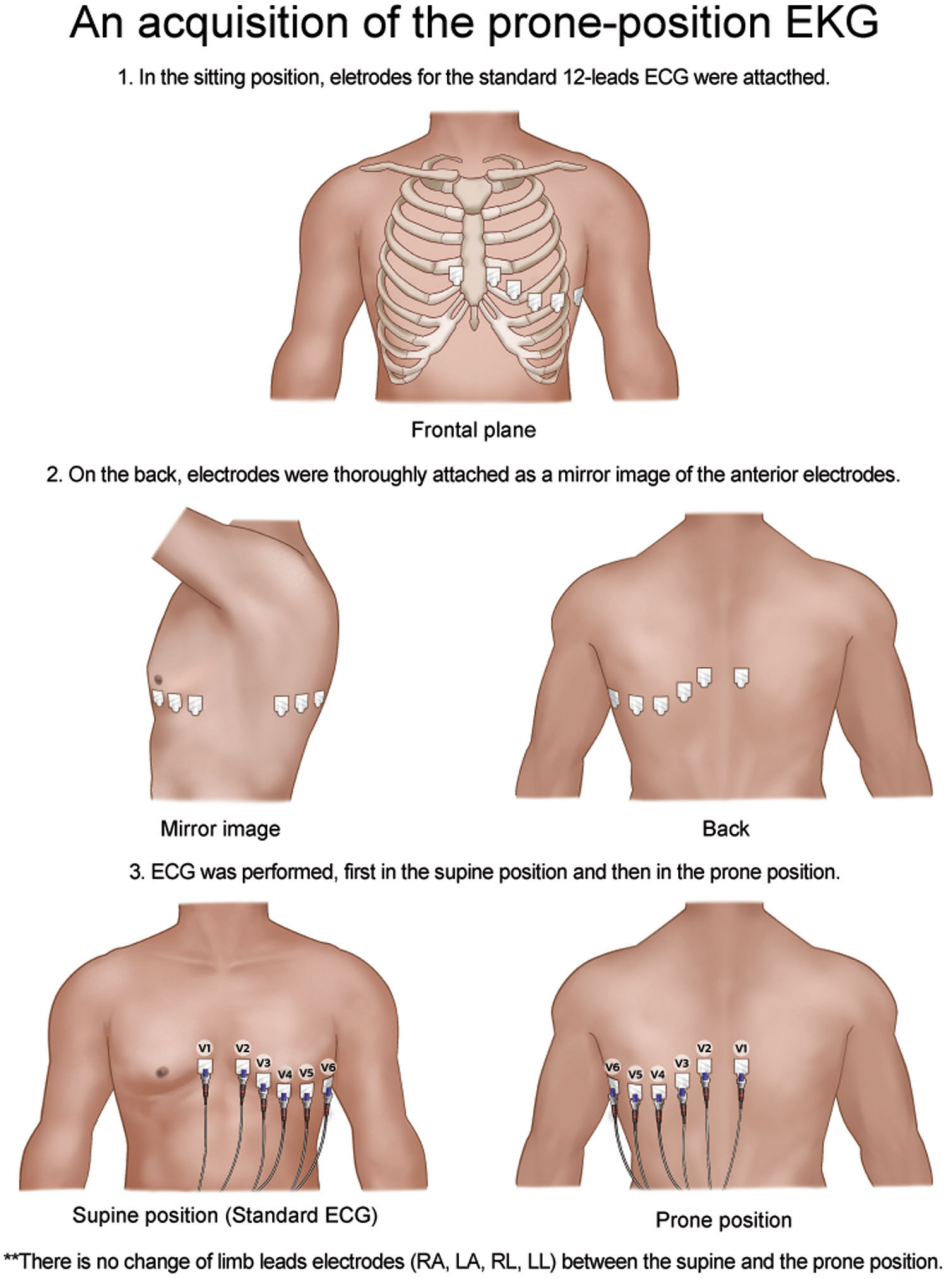
An acquisition of the standard and the prone ECG. The patient was seated, and standard 12‐lead ECG electrodes were applied following conventional guidelines. Electrodes on the patient's back were precisely arranged to create a mirror image of the front (chest) electrodes. The placement of the limb lead electrodes—RA (right arm), LA (left arm), RL (right leg), and LL (left leg)—remained unaltered during the transition.

1. The patient was initially seated, and standard 12‐lead ECG electrodes were placed according to the standard recommendations [22]. This involved the attachment of the electrodes to the patient's limbs and precise locations on the chest for the collection of standard ECG data (Figure 1).
2. Electrode placement for prone ECG on the patient's back was meticulously positioned to reflect a mirror image of the anterior (chest) electrodes. This mirroring was essential to maintain the consistency of the lead perspectives when the patient was turned to the prone position (Figure 1).
3. The ECG recording was first carried out with the patient in the supine position. This serves as a standard ECG reading. Subsequently (a few minutes later), the patient is turned to the prone position, taking care to ensure that the limb lead electrodes (RA—right arm, LA—left arm, RL—right leg, LL—left leg) remain unchanged during the transition (Figure 1).

#### 2.4.2 ECG Readings

ECG acquired in both the standard and prone positions were subjected to independent interpretation by two board‐certified cardiologists. To mitigate bias, the cardiologists were blinded to both the position in which the ECG was obtained and the identities of the patients. The software provided automated measurements for heart rate, QRS axis, PR interval, QRS duration, and the heart rate‐corrected QT interval (QTc). Subsequently, cardiologists reviewed and integrated these data with other relevant findings to formulate the final diagnosis. Each cardiologist's analysis was documented separately to ensure the integrity of the blinding process. In the event of a discrepancy in the diagnostic conclusions, a third cardiologist was consulted. This adjudicator was tasked with reviewing the conflicting interpretations and rendering a final decision, which was then incorporated into the data set for analysis.

#### 2.4.3 Clinical Severity Assessment

1. Saturation of peripheral oxygen (SpO_2_)/Fraction of inspired oxygen (FiO_2_) ratio (SF ratio):
  We used the SF ratio as a tool for the assessment of respiratory function, particularly in the context of acute respiratory distress syndrome (ARDS) and degrees of respiratory complications, in COVID‐19 patients in this study. SpO_2_ was measured using a pulse oximeter. FiO_2_ represented the percentage of oxygen in the air mixture that a patient was breathing. Room air is approximately 21% oxygen. Based on the findings of Brown et al., an SF ratio below 300 was indicative of significant hypoxemic respiratory failure in COVID‐19 patients [23]. Consequently, in our analysis, we adopted this threshold for statistical evaluation.
2. Acute Physiology and Chronic Health Evaluation II (APACHE II) score:
  In our study, we employed the APACHE II scoring system, utilizing a threshold score of greater than 12, to delineate the severity of COVID‐19 patients, particularly focusing on severe respiratory failure and other critical complications. This threshold was chosen based on the APACHE II scoring system's established role in predicting mortality and assessing severity in critical care settings [24].

### 2.5 Statistical Analysis

#### 2.5.1 Sample Size Calculation

The calculation followed the formula based on the study by David et al. [21], aimed at determining the difference in mean outcomes between two groups (ECG positions). The hypothesized difference in outcomes between the groups in our example, *µ*0 = 0.11 and *µa* = 0.0. The anticipated standard deviation in the population was set at 0.3. Additionally, the formula considered the Type I error rate (*α*), the probability of a false positive, which was set at 0.05, and the power (1‐*β*) of the study, set at 0.80, representing the probability of correctly rejecting the null hypothesis. The *Z*‐score (*z*) used, which corresponded to a 95% confidence interval, was 1.96. The computed analysis indicated a requisite sample size of 60 participants.

#### 2.5.2 Statistical Analysis

Quantitative variables were expressed as the mean ± standard deviation (SD) or median with interquartile range (IQR). Qualitative variables were delineated as frequencies and percentages. Intra‐subject comparison of continuous variables was facilitated by analysis of variance. The chi‐square test was employed to discern differences in categorical variables between patients with unfavorable outcomes versus those without. The Bland‐Altman plot and Pearson's correlation coefficient were utilized to determine the agreement and correlation of various ECG parameters in prone vs standard ECG. Univariate logistic regression analysis was conducted to investigate the associations between ECG parameters and clinical outcomes. A *p*‐value of less than 0.05 was considered indicative of statistical significance. Statistical analyses were executed using STATA software, version 10.1 (Texas, USA).

## 3 Results

### 3.1 Demographic and Clinical Data of the Study Population

During the study period, a total of 1918 patients were diagnosed with COVID‐19. For this study, 60 patients who were admitted to the specialized COVID‐19 treatment unit, were consecutively enrolled. Among the enrolled cohort, 36 patients (60%) were men. The mean age was 55.4 ± 16.8 years old. Comorbid conditions were prevalent, with diabetes mellitus (35%), and hypertension (32%). Initial presentation of hypoxemia was found in 19 patients (32%). The mean systolic blood pressure was 133 ± 19 mmHg, and the mean diastolic blood pressure was 82 ± 11 mmHg. A notable finding was tachypnea (22 ± 5 breaths per minute). Chest X‐ray revealed abnormalities in 58 of 60 patients (97%). However, the initial peripheral oxygen saturation (SpO_2_) was within normal limits, averaging 96 ± 2% (Table 1). Laboratory analysis indicated elevated random plasma glucose levels (190.7 ± 114.0 mg/dL). D‐dimer levels, although measured in less than half of the study population (38%), were markedly elevated, 4470 ± 901 ng/mL. CRP levels, tested in all patients, were elevated, showing a mean of 30 ± 34 mg/L (Table 1).

**Table 1:** Baseline characteristics.

	*N* = 60
Age, years	55 ± 17
Men, *n* (%)	36 (60)
Weight, kg	71 ± 18
Height, cm	161 ± 18
Initial vital signs	
Temperature, degree Celsius	37.1 ± 0.9
SBP, mmHg	133 ± 19
DBP, mmHg	82 ± 11
RR, bpm	22 ± 5
HR, bpm	88 ± 13
SpO_2_, %	96 ± 2
Initial blood tests	
Hematocrit, %	38.9 ± 5.3
WBC counts, ×10^6^/L	9494 ± 4390
Platelet counts, ×10^6^/L	256 900 ± 108 099
Creatinine, mg/dL	1.15 ± 1.17
Blood glucose, mg/dL	190.8 ± 114.0
Sodium, mEq/L	136.6 ± 3.8
Potassium, mEq/L	4.5 ± 0.6
HCO_3,_ mEq/L	21.6 ± 3.7
CRP, mEq/L	30.0 ± 23.7
Multifocal lung lesion, *n* (%)	43 (71.7)
Systemic steroids treatment, *n* (%)	57 (95.0)
APACHE II score	7 ± 5
In‐hospital death, %	5.0 (8.4)
SF ratio	354 ± 125

Abbreviations: APACHE II scores, Acute Physiology and Chronic Health Evaluation II scores; CRP, C‐reactive protein; DBP, diastolic blood pressure; HCO_3_, sodium bicarbonate; HR, heart rate; RR, respiratory rate; SBP, systolic blood pressure; SF ratio, saturation of peripheral oxygen/fraction of inspired oxygen ratio; SpO_2_, saturation of peripheral oxygen; WBC, white blood cell count.

The mean APACHE II score of the patients was 7 ± 5. The mean SF ratio was calculated to be 354 ± 125. As for the hospitalization course, a subset of the cohort (five patients, 8.3%), exhibited progressive respiratory failure, necessitating the use of mechanical ventilation. Additionally, 12 patients (20%) experienced clinical deterioration warranting transfer to the intensive care unit (ICU). Regarding patient survival, 57 patients (95%) were successfully discharged from the hospital. However, at the 6‐month follow‐up, the number of surviving patients decreased to 54 (90%). The causes of mortality during this period included acute myocardial infarction, acute pulmonary embolism, stroke, bacterial pneumonia, and hemophagocytic lymphohistiocytosis, cumulatively accounting for six deaths at the 6‐month mark (Table 1).

### 3.2 Comparisons of the ECG Parameters Between the Standard and the Prone ECG

In the prone position, an increase in heart rate was observed, with mean values rising from 74 ± 17 bpm in standard ECG to 76 ± 16 bpm in prone ECG (mean difference: 2.100 (95% CI 0.471–3.729, *p* = 0.012). The mean QRS axis also exhibited a minor increase from 30 ± 37 degrees to 32 ± 44 degrees. Regarding the T wave axis, a slight decrease was noted, from 33 ± 36 degrees in standard ECG to 31 ± 45 degrees in prone ECG. A more notable change was seen in the P wave axis, which decreased from 44 ± 23 degrees to 37 ± 31 degrees. Minimal variations were observed in the duration of the QRS complex (99 ± 38 to 96 ± 13 ms) and the QTc interval (447 ± 71 to 441 ± 32 ms). The PR interval showed a slight reduction from 162 ± 25 ms 160 ± 33 ms, in standard and prone ECG, respectively. However, except for heart rate, no observed differences in the other parameters achieved statistical significance.

### 3.3 Correlation Analyses of ECG Parameters

Agreement limits for ECG parameters between prone and standard positions ranged from −14.710 to 96.243, with Pearson's correlation coefficients varying from −0.181 to 0.927, indicating diverse degrees of linear relationships. Specifically, heart rate exhibited a strong positive correlation (*r* = 0.927, *p* < 0.001), with limits of agreement from −14.710 to 10.510. The PR interval also showed a moderately strong positive correlation (*r* = 0.784, *p* < 0.001), with limits of agreement from −39.424 to 43.642. Conversely, QRS duration revealed a weak negative correlation (*r* = −0.181, *p* = 0.168), with broad limits of agreement (−81.567 to 88.600). QTc and the P wave axis presented moderate positive correlations (*r* = 0.639 and *r* = 0.559, respectively, *p* < 0.001), with respective limits of agreement. Notably, the QRS axis also displayed a strong positive correlation (*r* = 0.894, *p* < 0.001). On the other hand, the T wave axis demonstrated a weak positive correlation with a Pearson's coefficient of 0.324 (*p* = 0.012) with the limits of agreement ranging considerably, from −93.022 to 96.243. These results highlight the varying degrees of agreement and correlation for ECG parameters in different ECG positions, with the most significant correlations observed in heart rate and QRS axis, contrasting with the weak negative correlation in QRS duration (Supporting Information S1: Figure S1).

### 3.4 Correlation Analyses of ECG Diagnosis

ECG diagnostic categorization for analysis was derived from the consensus of two independent cardiologists who were blinded to the ECG position. The diagnoses encompassed a spectrum of cardiac rhythms and pathologies, including normal sinus rhythm, sinus tachycardia, sinus bradycardia, atrial fibrillation, premature ventricular complexes, first‐degree AV block, acute ST‐segment elevation myocardial infarction (STEMI), acute non‐ST‐segment elevation myocardial infarction (NSTEMI), historical myocardial infarction (old MI), early repolarization, bundle branch block (BBB), intraventricular conduction delay, and left ventricular hypertrophy (LVH). Concordance between standard and prone ECG diagnoses was measured at 43.3%, with a Cohen's Kappa coefficient of 0.296 (95% CI: 0.244–0.379, *p* < 0.001) (Table 2).

**Table 2:** The diagnosis from the standard and the prone ECG.

Diagnosis	Standard ECG *n* (%)	Prone ECG *n* (%)
Normal sinus rhythm	21 (35)	27 (45)
Sinus tachycardia	2 (3.3)	3 (5)
Sinus bradycardia	6 (10)	8 (13.3)
Atrial fibrillation	3 (5)	2 (3.3)
Premature ventricular complex	1 (1.7)	1 (1.7)
First degree AV block	3 (5)	3 (5)
STEMI	1 (1.7)	0
NSTEMI	0	1 (1.7)
Old MI	1 (1.7)	5 (8.3)
Early repolarization	5 (8.3)	3 (5)
RBBB	10 (16.6)	4 (6.6)
Intraventricular conduction delay	1 (1.7)	1 (1.7)
LV hypertrophy	6 (10)	1 (1.7)

Abbreviations: AV, atrioventricular; LV, left ventricular; MI, myocardial infarction; NSTEMI, non‐ST‐segment elevation myocardial infarction; RBBB, right bundle branch block; STEMI, ST‐segment elevation myocardial infarction.

An intriguing finding of our study was the increase in the number of diagnoses of old MI when comparing standard ECGs to those obtained in the prone position. Specifically, the number of cases rose from one with the standard ECG to five with the prone‐position ECG. This increase can be attributed to the appearance of Q waves in leads V1 and V2 in the prone position for some patients, likely due to the altered cardiac axis associated with the prone positioning (Supporting Information S1: Table S1).

### 3.5 The Association of ECG Parameters From the Prone and Standard Positions and the Severity of COVID‐19 Patients

#### 3.5.1 Association of ECG Parameters With SF ratio < 300

1. Standard ECG:
  The analysis revealed that patients with a QTc interval > 460 ms had an odds ratio (OR) of 1.08 (95% CI: 0.28–4.20, *p* > 0.999) for having an SF ratio of < 300. For every 1 bpm increase in HR, the OR was 0.97 (95% CI: 0.94–1.00, *p* = 0.086). This finding suggests a slight, albeit not statistically significant. The presence of ST segment changes (either elevation or depression) was associated with an OR of 1.21 (95% CI: 0.34–4.31, *p* = 0.755) for an SF ratio of < 300. T wave inversion was associated with an OR of 0.47 (95% CI: 0.13–1.69, *p* = 0.241). In summary, while there were associations observed between certain ECG parameters and an SF of < 300, none of these associations reached statistical significance.
2. Prone ECG:
  A QTc interval greater than 460 ms was associated with an OR of 0.91 (95% CI: 0.26–3.12, *p* = 0.876). For every increase of 1 bpm in HR, the OR was 0.96 (95% CI: 0.93–0.99). This finding indicates a statistically significant decrease in the odds of having an SF Ratio of < 300 with each incremental increase in HR, as evidenced by the *p*‐value of 0.046. The presence of ST segment changes was associated with an OR of 0.76 (95% CI: 0.18–3.32, *p* > 0.999). The presence of T wave inversion was associated with a notably higher OR of 6.90 (95% CI: 0.82–58.21, *p* = 0.078), suggesting an increased likelihood of having an SF Ratio of < 300. However, this association did not reach statistical significance. In summary, the only statistically significant association observed was the decreased likelihood of having an SF Ratio of < 300 with each increase in HR reported in the prone ECG. The other ECG parameters, namely QTc greater than 460 ms, ST segment changes, and T wave inversion did not show significant associations.

#### 3.5.2 Association of ECG Parameters With APACHE II Score > 12

1. Standard ECG:
  A QTc interval over 460 ms was associated with an OR of 4.22 (95% CI: 0.79–22.53, *p* = 0.108). For every one beat per minute increase in HR, the OR was 1.04 (95% CI: 0.99–1.09, *p* = 0.141). The presence of ST segment changes was associated with a notably higher OR of 6.52 (95% CI: 1.24–34.28, *p* = 0.034), indicating a significantly increased likelihood of having an APACHE II Score > 12. T wave inversion showed an OR of 1.01 (95% CI: 0.18–5.80, *p* > 0.999). In summary, the most notable finding was the significant association between ST segment changes and an APACHE II Score > 12. The associations with a prolonged QTc interval, increased HR, and T wave inversion, while suggestive of trends, did not reach statistical significance.
2. Prone ECG:
  A QTc interval exceeding 460 ms was strongly associated with an APACHE II Score > 12, as indicated by an OR of 10.75 (95% CI: 1.82–63.64, *p* = 0.008). Each increase of 1 bpm in HR was associated with an OR of 1.03 (95% CI: 0.98–1.08). However, this association did not reach statistical significance (*p* = 0.216). The presence of ST segment changes showed an OR of 2.25 (95% CI: 0.37–13.67, *p* = 0.330), indicating a potential increase in the likelihood of an APACHE II Score > 12. However, this finding was not statistically significant. T wave inversion was associated with an OR of 0.51 (95% CI: 0.09–3.06, *p* = 0.602). In summary, the most significant finding was the association between a QTc interval greater than 460 ms and an APACHE II Score above 12. Other ECG parameters, such as HR, ST segment changes, and T wave inversion, did not demonstrate statistically significant associations in this context.
  The overall results indicate varying degrees of association between ECG parameters and both the SF ratio < 300 and APACHE II score > 12 in standard and prone positions. Statistical analysis revealed a significant negative correlation between HR in the prone position and an SF ratio < 300. Conversely, a significant positive correlation was observed between an APACHE II score > 12 and ST‐segment alterations in standard ECGs, as well as a QTc > 460 ms in the prone ECG. No other associations demonstrated statistical significance (Tables 3 and 4).

**Table 3:** The association of ECG parameters from the prone and standard positions and the SpO_2_/FiO_2_ ratio < 300.

	OR (95% CI)	*p*‐value
Standard ECG		
QTc > 460 ms	1.08 (0.28, 4.20)	> 0.999
HR (every 1 bpm)	0.97 (0.94, 1.00)	0.086
ST segment change	1.21 (0.34, 4.31)	0.755
T wave inversion	0.47 (0.13, 1.69)	0.241
Prone ECG		
QTc > 460 ms	0.91 (0.26, 3.12)	0.876
HR (every 1 bpm)	0.96 (0.93, 0.99)	0.046
ST segment change	0.76 (0.18, 3.32)	> 0.999
T wave inversion	6.90 (0.82, 58.21)	0.078

Abbreviations: CI indicates confidence interval; ECG, electrocardiogram; FiO_2_, fraction of inspired oxygen; HR, heart rate; OR, Odd ratio; QTc, QT interval corrected for heart rate; SpO_2_, saturation of peripheral oxygen.

**Table 4:** The association of ECG parameters from the prone and standard positions and the APACHE II score > 12.

	OR (95% CI)	*p*‐value
Standard ECG		
QTc > 460 ms	4.22 (0.79, 22.53)	0.108
HR (every 1 bpm)	1.04 (0.99, 1.09)	0.141
ST segment change	6.52 (1.24, 34.28)	0.034
T wave inversion	1.01 (0.18, 5.80)	> 0.999
Prone ECG		
QTc > 460 ms	10.75 (1.82, 63.64)	0.008
HR (every 1 bpm)	1.03 (0.98, 1.08)	0.216
ST segment change	2.25 (0.37, 13.67)	0.330
T wave inversion	0.51 (0.09, 3.06)	0.602

Abbreviations: APACHE II, acute physiology and chronic health evaluation II; CI, confidence interval; ECG, electrocardiogram; HR, heart rate; OR, odd ratio; QTc, QT interval corrected for heart rate.

## 4 Discussion

Cardiac positioning induced by body posture has been found to impact ECG parameters, particularly the electrical axis, wave amplitude, and ST segment. While earlier research covered a range of positions like supine, sitting, and standing [25], recent studies have increasingly focused on the prone position's effects on ECG recordings [4, 5, 6, 16, 17, 18, 19, 20]. The present study showed that prone positioning elicited minor increases in HR and QRS axis deviation, suggesting position‐dependent cardiac electrical alterations. Additionally, ECG parameters varied in their agreement and correlation, with heart rate exhibiting a strong positive correlation and QRS duration displaying a weak negative correlation. The fair concordance in ECG diagnoses found in this study necessitates refinement in interpretative consistency. Additionally, a significant correlation between QTc prolongation of the prone ECG and severity of COVID‐19, as measured by APACHE II scores, was observed, underscoring the ECG's prognostic relevance in ARDS management.

In our study, prone‐positioned ECGs demonstrated a modest but statistically significant elevation in mean heart rate, increasing from 74 ± 17 bpm in standard ECGs to 76 ± 16 bpm. This finding is consistent with previous research in both ARDS patients [17, 20] and healthy adults [19]. Additionally, we noted a slight alteration in the QRS axis, shifting from 30 ± 37 to 32 ± 44 degrees. This deviation diverges from the results observed in a study involving non‐COVID ARDS adults [17] but aligns with findings from a study on COVID‐19 ARDS patients [20]. Other parameters, including QRS duration, QTc, and PR interval, showed nonsignificant variations. Contrary to previous studies reporting a notable decrease in prone ECG's QRS duration, our study observed only a minimal reduction compared to supine ECG. The underlying mechanisms for these observations and the variability in study outcomes are not fully elucidated. However, two hypotheses are suggested: first, the prone position's known effect on autonomic modulation, may significantly influence heart rate and cardiac repolarization [26]. Second, morphological changes in the heart during prone positioning, leading to a more globular shape with greater thoracic wall contact, might explain the alterations in QRS duration and QTc [27].

Agreement limits across ECG parameters revealed broad variability, with Pearson's correlations ranging from −0.181 to 0.927, underscoring diverse linear associations between standard and prone positions. The strong positive correlation in heart rate suggests that heart rate measurements are relatively consistent between prone and standard positions. This implies that heart rate can be a reliable parameter to monitor across different positions in clinical settings. The PR interval also showed a moderately strong positive correlation. However, the weak negative correlation in QRS duration indicates that these parameters might be influenced by positional changes. This should be considered during ECG analysis.

Diagnostic categorizations for ECGs, collaboratively established by two cardiologists, covered from normal rhythms to complex pathologies. Concordance between standard and prone ECGs demonstrated moderate agreement (43.3%), with a Cohen's *κ* of 0.296 (95% CI: 0.244–0.379, *p* < 0.001), suggesting fair but limited consistency. Although there was good agreement in cardiac rhythm diagnoses, significant discrepancies were noted in old myocardial infarction and right BBB cases. These findings, in line with previous research, indicated altered ECG patterns in prone positions that could affect the detection of myocardial infarction and sensitivity to RBBB [17, 21]. Given the moderate Kappa value, clinical reliance on prone ECG readings, particularly for MI diagnosis, warrants careful interpretation within the specific clinical context.

In terms of disease prognosis, our study revealed significant correlations between prone ECG parameters and COVID‐19 severity. A notable finding was the association of prolonged QTc intervals with elevated APACHE II scores above 12 (RR = 10.75, 95% CI: 1.82–63.64, *p* = 0.008). Prone positioning is a therapeutic intervention for ARDS, known for its positive effects on oxygenation [6, 28]. This position promotes dorsal lung recruitment and more even ventilation distribution, which can reduce respiratory rate and improve pulmonary function [29]. The impact on cardiac function, especially in patients with hypoxemic respiratory failure, is less understood. Studies have shown varying cardiac responses in different patient populations and volume statuses [30]. For example, in patients with sufficient cardiac reserve, prone positioning can increase cardiac preload and decrease right ventricular afterload. This effect, however, is not seen in patients lacking preload reserve [30]. In critically ill patients, particularly in intensive care units [31], QTc prolongation is relatively common, and its incidence increases with the duration of hospitalization, serving as a mortality predictor [32]. Factors contributing to QTc prolongation include gender, age, electrolyte imbalances, bradycardia, and existing cardiac conditions [31]. In our study, a correlation was observed between prolonged QTc intervals in the prone position and elevated APACHE II scores, indicating increased severity in COVID‐19 and ARDS patients. This correlation, however, was not observed in standard positioning. The specific mechanisms behind prolonged QTc intervals in patients with high APACHE scores are not fully clear, but it is theoretically expected that prone positioning should have a positive effect on hemodynamics and oxygenation.

In our study, we observed that each 1 bpm increase in heart rate was associated with a decrease in ARDS severity, as indicated by SpO_2_/FiO_2_ ratios below 300 (RR = 0.96, 95% CI: 0.93–0.99, *p* = 0.046). This finding aligns with previous research showing elevated heart rates in prone ECG compared to supine ECG in ARDS patients [17, 20]. This elevation might reflect the impact of prone positioning on hemodynamics, potentially resulting in increased cardiac index or decreased pulmonary vascular resistance in some patients [30]. Therefore, elevated heart rates in a prone position could suggest a preserved physiological response, indicative of lower cardiopulmonary system severity. However, this interpretation remains hypothetical, and the underlying mechanisms require further elucidation.

Furthermore, ST‐segment changes in standard ECG emerged as strong predictors of higher APACHE II scores (RR = 6.52, 95% CI: 1.24–34.28, *p* = 0.034). Previous studies have identified that specific ECG abnormalities, such as ST depression, T‐wave inversion, and ST‐T changes at admission, are closely linked to the severity of COVID‐19 infection. These findings suggest that ECG can be a valuable tool in assessing the clinical status of COVID‐19 patients. Additionally, ST‐segment elevation in COVID‐19 patients, irrespective of whether it is attributed to obstructive or nonobstructive coronary artery disease, has been associated with a poor prognosis. This highlights the critical nature of ST‐segment changes in this patient population and underscores the need for careful cardiovascular evaluation and management [33, 34]. We observed a lack of correlation between ST‐segment changes in prone ECGs and the severity of COVID‐19 infection which could be due to the unique impact of prone positioning on cardiac electrophysiology, particularly affecting the anterior precordial leads. Prone positioning may induce changes in the ST segment that do not necessarily reflect underlying cardiac pathology but are rather artifacts of the position itself [21].

This study's single‐center design may limit the generalizability of its findings, and the small number of cases could reduce the ability to detect a significant association between a QTc interval > 460 ms and elevated APACHE II scores in the standard ECG group. Additionally, the absence of serial ECGs over time restricts our conclusions to associations rather than causal relationships between ECG parameters and COVID‐19 severity. We also did not account for other factors affecting heart rate, such as stress or discomfort from prone positioning, and the lack of echocardiographic data limits our understanding of hemodynamic changes in this context.

## 5 Conclusions

This study demonstrates the influence of the prone position on ECG parameters, revealing an increase in heart rate and QRS axis deviation indicative of position‐dependent cardiac electrophysiological changes. It is among the first to establish an association between prolonged QTc intervals in prone ECGs and increased APACHE II scores in COVID‐19 patients with moderate ARDS. This finding suggests the potential utility of prone ECG, especially QTc evaluation, in assessing COVID‐19 severity. However, further investigation is needed to fully understand the mechanisms and clinical significance of these observations.

## Conflicts of Interest

The authors declare no conflicts of interest.

## Supporting information

Supporting information.

## Data Availability

The data that support the findings of this study are available from the corresponding author upon request.
