# Supplementary material for: Comparison of Standard and Prone‐Position Electrocardiograms in COVID‐19 Patients With Pulmonary Complications: Correlations and Implications
Source: Clin Cardiol. 2024 Sep 30;47(10):e70024. doi: 10.1002/clc.70024 (PMC11440021; doi:10.1002/clc.70024)
Supplement: Supplementary file 1 — Supporting information. [file CLC-47-e70024-s001.docx]

**Supplementary material**

**Title: Comparison of standard and prone-position electrocardiograms in COVID-19 patients with pulmonary complications: Correlations and implications**

Pattarapong Makarawate, M.D.^1^, Krissanachai Chimtim, M.D.^2^, Thapanawong Mitsungnern, M.D.^2^, Pariwat Phungoen, M.D.^2^, Supap Imoun, M.N.S.^3^, Piroon Mootsikapun, M.D.^1^, Thanat Tangpaisarn, M.D.^2^, Praew Kotruchin, M.D., Ph.D.^2^

^1^ Department of Internal Medicine, Faculty of Medicine, Khon Kaen University, Khon Kaen, Thailand.

^2^ Department of Emergency Medicine, Faculty of Medicine, Khon Kaen University, Khon Kaen, Thailand.

^3^ Accident and Emergency Stroke Unit, Srinagarind Hospital, Faculty of Medicine, Khon Kaen University, Khon Kaen, Thailand.

**Supplement figure**


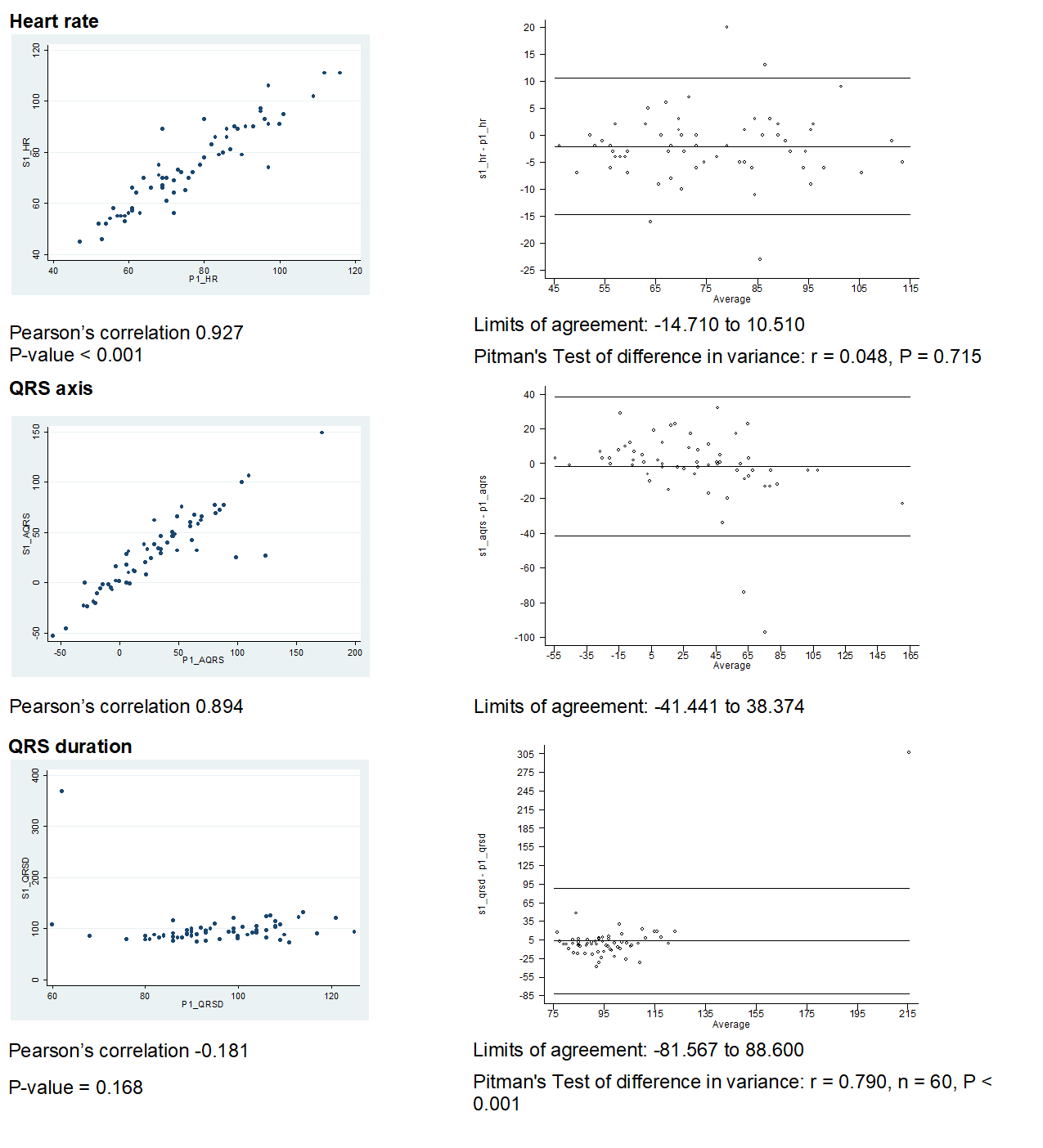


***The Bland–Altman plots and Pearson’s correlations for Heart rate, QRS axis, and QRS duration between the standard and the prone ECG*** Heart rate exhibited a strong positive correlation (r = 0.927, P < 0.001), the PR interval also showed a moderately strong positive correlation (r = 0.784, P < 0.001), QRS duration revealed a weak negative correlation (r = -0.181, P = 0.168), QTc and P wave axis presented moderate positive correlations (r = 0.639 and r = 0.559, respectively, P < 0.001). Notably, the QRS axis also displayed a strong positive correlation (r = 0.894, P < 0.001). On the other hand, the T wave axis demonstrated a weak positive correlation with a Pearson’s coefficient of 0.324 (P = 0.012).

**Supplement table:** Comparison of ECG diagnoses between supine and prone positions: consistency in case no.1, and discrepancy in case no.2

| Case examples | Clinical presentation | Underlying | Supine ECG and diagnosis | Prone ECG and diagnosis |
| --- | --- | --- | --- | --- |
| Case no.1 with consistent diagnosis between supine and prone ECGs | 70-year-old female with fever and dyspnea with poorly define unilateral ground glass pattern on chest X-ray | none | 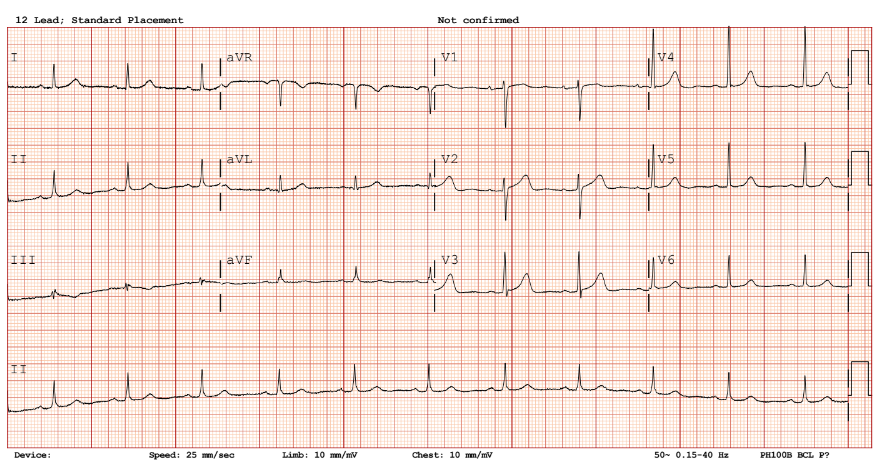 | 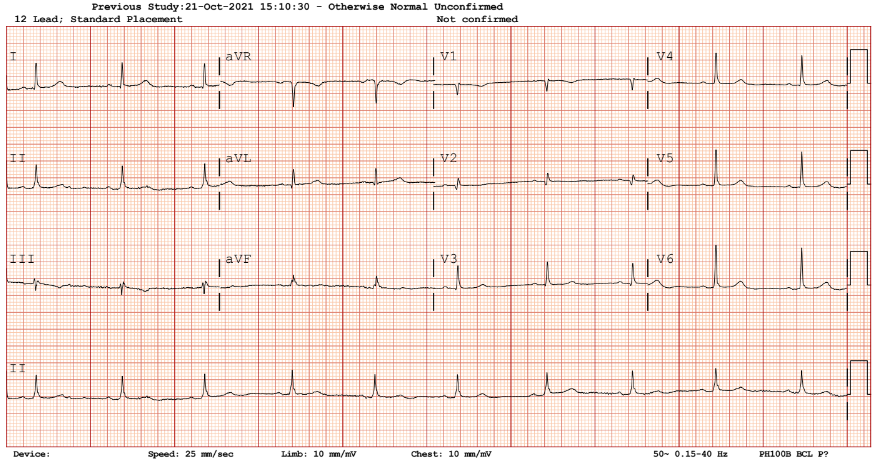 |
|  |  |  | Normal sinus rhythm | Normal sinus rhythm |
| Case no.2 with discrepant diagnoses between supine and prone ECGs | 50-year-old male with fever and tachypnea with multifocal opacities on chest X-ray | none | 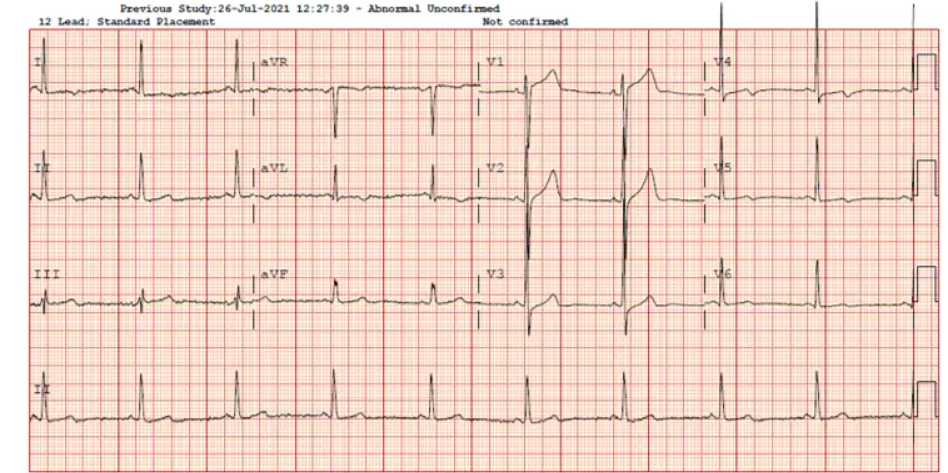 | 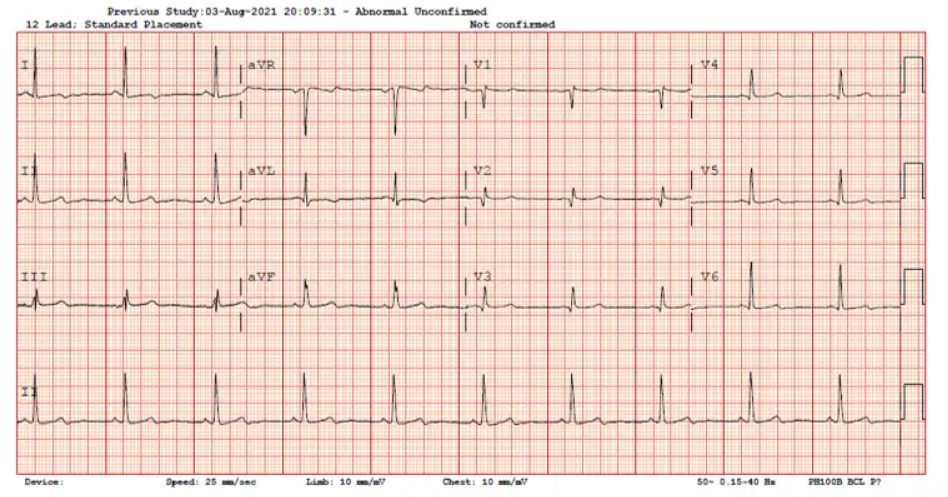 |
|  |  |  | Sinus bradycardia with LVH | Sinus bradycardia with tall R in V1 V2, and Q in V1 V2 (old myocardial infarction) |
